# Lower Limb Biomechanical Observations in Hypermobile Children: An Exploratory Case—Control Study

**DOI:** 10.3390/ijerph22121776

**Published:** 2025-11-24

**Authors:** Muhammad Maarj, Verity Pacey, Louise Tofts, Antoni Fellas, Matthew Clapham, Andrea Coda

**Affiliations:** 1 School of Health Sciences, College of Health, Medicine and Wellbeing, The University of Newcastle, Ourimbah, NSW 2258, Australia; 2 Narrabeen Sports Medicine Centre, Sydney Academy of Sport, Sydney, NSW 2101, Australia; 3 Department of Health Sciences, Macquarie University, Sydney, NSW 2109, Australia; 4 Equity in Health and Wellbeing Research Program, Hunter Medical Research Institute, Newcastle, NSW 2305, Australia; 5 Hunter Medical Research Institute, Newcastle, NSW 2305, Australia

**Keywords:** biomechanics, foot posture, hypermobility, musculoskeletal condition

## Abstract

Generalised joint hypermobility (GJH) describes an excessive range of joint movement and is associated with increased musculoskeletal injury risk, joint pain, and instability. This study compares lower limb biomechanical characteristics between children with and without GJH. Children aged 5–18 years with GJH (Beighton score ≥ 6/9 pre-puberty, ≥5/9 post-puberty) were age- and sex-matched with controls (Beighton score ≤ 2/9). Biomechanical measures included internal hip rotation, quadriceps (Q) angle, tibial torsion, ankle range of motion (ROM), and foot posture index (FPI). Wilcoxon rank sum test and chi-square were used to assess group differences. Fifty-two participants (median age 11 years, 69% females) included 27 children with GJH and 25 healthy children. Internal hip rotation, Q-angle, ankle ROM and FPI were significantly higher for children with GJH (*p* < 0.001) than healthy peers. While tibial torsion showed no difference in males, females had greater internal tibial torsion [median difference: right −4° (95%CI:−7,−2), *p* = 0.002; left −5° (95%CI:−7,−1), *p* = 0.010]. The largest differences were in ankle ROM [median difference: right 9° (95%CI:7,12); left 9° (95%CI:6,12)]. Children with GJH present different biomechanical measures than non-GJH peers. Further research into the clinical relevance of ROM at the hip, ankle and foot for children with GJH which are movement planes not assessed in Beighton score is warranted.

## 1. Introduction

Generalised joint hypermobility (GJH) is clinically identified as excessively high range of motion (ROM) in multiple joints [1]. GJH is measured using the Beighton score [2] which assesses only a single joint (the knee) in the lower limb. Laxity, which is often referred to as mechanical instability [3], is passive joint movement outside usual physiological limits during motion and may lead to subluxation or dislocation [4]. While laxity often accompanies GJH it is distinct and not measured using the Beighton score [4,5].

Approximately one-fifth of children with GJH develop symptoms [6], including activity-related joint pain, soft tissue injuries, and/or recurrent instability of joints [7]. Lower limb pain is the most common complaint from by this cohort [8]. Children with GJH have been reported to have more ligament injuries [8,9], joint instability episodes [8,10], and risk of increased musculoskeletal injuries [11]. In addition, evidence suggests that children with GJH often present with complaints related to their lower limbs and experience functional difficulties such as pain [12,13], reduced muscle strength [10,14], and fatigue [15] that can significantly affect their mobility, negatively impacting participation and resulting in poor health outcomes with reduced quality of life [16,17,18]. The literature further suggests that the pain, fatigue, and quality of life experiences of children with GJH are similar to those of children with chronic pain seeking care through physical therapy [19].

While the Beighton score is widely used for assessing general joint hypermobility, specific measures for the ankles or feet are not included. This exclusion from the Beighton score has been highlighted as a limitation in accurately assessing hypermobility present in lower limbs in the paediatric population [2,20] and can be problematic, especially since the feet and ankles are frequently identified as major sources of discomfort and symptoms in children with hypermobility [21,22]. Research suggests that these areas are particularly vulnerable due to the biomechanical demands placed on them, and neglecting to assess them may overlook a critical aspect of hypermobility in children. For instance, studies have shown that abnormal foot posture and ankle range in adults [23] and children with hypermobility [24,25] have been associated with altered gait kinematics. In young dancers, aged 7–12 years, the subtalar joint axis was medially deviated in dancers with GJH (Beighton score ≥ 7/9) and associated with increased pronation [26]. Including ankle and foot assessments could provide a more comprehensive understanding of how hypermobility impacts lower limb mechanics.

Clinical reports suggest that adults with GJH commonly present with muscle weakness, joint instability, internally rotated hip position, tibial torsion and excessively pronated feet [27]. Hawke et al. 2016 reported that there is a correlation between a high foot posture index (FPI) score and increased joint hypermobility [28]. Furthermore, the specific scores for analysis of ROM for other lower extremity joints (foot, ankle dorsiflexion, hip external rotation, hip abduction, and hip extension) is higher among dancers with GJH compared to dancers without GJH, in particular young dancers aged 8 to 10 years [29].

Considering the greater range of motion in individuals with GJH, it is important that clinicians are aware of any relevant biomechanical characteristics in addition to joint hypermobility when planning management strategies. Rotational measures, including internal hip rotation and tibial torsion, are important biomechanical features to consider. Higher internal hip rotation has been associated with functional problems, such as in-toeing gait [30] as well as pronation [31]. In a recent study of children with increased femoral anteversion (IFA), a condition which is commonly seen with hypermobility [32], lower-extremity function worsened compared to healthy peers with a higher frequency of falling as the internal hip rotation angle increased in children with IFA [33]. Another rotational measure to consider is internal tibial torsion, a major cause for development of in-toeing gait which can have a detrimental effect on muscle function [34] by producing joint overload with subsequent patellofemoral dysfunction [35].

Since the risk of lower limb joint injuries is high in children and young adults with GJH, especially during sporting activities [11,36,37,38,39], the quadriceps angle (Q-angle), ankle ROM and FPI measures are also important biomechanical assessments to consider in this cohort. The Q-angle is defined as the angle formed between the anatomical landmarks of mid-point of the patella, the centre of tibial tubercle and the anterior superior iliac spine [40]. As an indicator of extensor mechanism dysfunction, Q-angle has been associated with anterior knee pain [41], patellofemoral pain syndrome [42] and knee osteoarthritis [43,44]. Furthermore, lower extremity biomechanics has been found to be impacted by excessive Q-angle, which influences patterns of stress on patella cartilage during repetitive activities, resulting in knee injuries [45,46].

Ankle ROM is a further biomechanical measure to consider since the ankle and knee joint together are predisposed to a higher degree of strain as they have weight-bearing and motor functions. Hypermobility of the ankle joint may result in the proximal transfer of forces at the foot [47], increasing the risk of injuries in children with GJH, such as ankle sprains [22]. Lower limb alignment is additionally influenced by foot posture in children with GJH, where greater FPI scores are reported for children and adolescents with a higher Beighton score [28,48]. Children with GJH frequently experience poor dynamic balance and impaired gait due to increased joint ROM, which may lead to overuse injuries [49,50]. The balance and gait patterns can be improved by wearing foot orthotics [49,51,52]. Therefore, FPI assessment is an important additional biomechanical measure to consider when planning physical therapy strategies to reduce the risk of lower extremity injuries in children with GJH.

Collectively, previous studies support the importance of common biomechanical measures, including internal hip rotation, tibial torsion, Q-angle, ankle ROM, and foot posture measurements. While assessment should be tailored to the presenting problems, these biomechanical measures may be considered to inform physical therapy strategies to improve gait patterns and prevent joint injuries as an integral part of clinical assessment of children with GJH.

Therefore, the purpose of this study was to clinically assess GJH and laxity in hip internal rotation, Q angle, tibial torsion, together with the foot and ankle ROM, in children with GJH compared to non-hypermobile controls and to determine whether there is a difference between hypermobile and non-hypermobile children. In addition, differences in biomechanical measures between sexes and age groups were explored. To support our research objective, we propose that there is a marked variation in biomechanical observations when comparing children with hypermobility to those without this condition.

## 2. Materials and Methods

### 2.1. Study Design

This study was a cross-sectional age and sex-matched case–control design approved by the University of Newcastle’s Human Research Ethics Committee (HREC, Ref no. H-2020-0387) and conducted in accordance with the National Statement on Ethical Conduct of research in Australia. All adolescent participants and caregivers for younger children provided signed informed consent.

Recruitment of children with GJH was undertaken from Narrabeen Sports and Exercise Medicine Centre (NSEMC) at the Sydney Academy of Sport and Recreation, Narrabeen, NSW. While the majority of the non-hypermobile participants, as the control group, were recruited from non-symptomatic physiotherapy class attendees at the Sydney Academy of Sport and Recreation and non-symptomatic attendees to NSEMC, team sports, and local activity classes. Participants who met the inclusion criteria for this study (Table 1) were provided with participation information.

### 2.2. Data Collection

Age, sex, Beighton score, height, weight, and body mass index (BMI) were collected to describe the study population. The study protocol was finalised before the publication of the 2023 paediatric diagnostic framework; therefore, the Beighton score cut-offs used to identify generalised joint hypermobility (the hypermobile group) were ≥6/9 for prepubertal children and ≥5/9 for pubertal participants. The design of the data collection process took into account the importance of completing the data collection within a short time in order to promote uptake of this study.

The Beighton score was used to identify the paediatric population affected with GJH [2]. Each participant was allocated one point for each of the following for a total of 9 points: the ability to hyperextend the elbows and knees on each side to greater than +10°, the ability to touch the tip of the thumb to the forearm for each side, the ability of the little finger on each side to extend beyond 90° at the metacarpophalangeal joint, and the ability to touch the floor with both hands while keeping the knees straight. The participants performed each task within their own comfort level [8].

During the study visit, a registered podiatrist (MM) conducted a set of standard biomechanical tests that are commonly used in clinical podiatric practice as described below (Table 2). These measures have previously shown moderate to high intrarater and interrater reliability (ICC: 0.82–0.99) in children aged 9 to 18 years [53]. For the ROM measurements a standard goniometer (Product Code: GON30R Company: total patient care Goniometer Plastic 360 Deg Range, Sydney, Australia) 30-centimetre plastic 180° degrees which is a validated and reliable clinical tool for passive range of motion of the lower extremity was used [54,55,56]. Measurements were all repeated three times to reduce measurement bias and increase accuracy, with values averaged to calculate the mean value.

#### 2.2.1. Hip Internal Rotation ROM (Craig’s Test)

To assess biomechanical alignment of lower extremities the femoral anteversion test or Craig’s test based on evaluation of hip rotation was performed [57]. This test has been validated with reported high intrarater and interrater reliability (ICC > 0.81) in healthy children and adolescents [58]. The passive hip internal rotation was measured with the participant in prone position at a neutral hip position with the knee at 90° flexion. The internal rotation was determined using Craig’s test to measure the angle of rotation with the greater trochanter prominence as a reference landmark by handheld standard goniometer. This measure has been shown to have good reliability with Computed Tomography imaging technique in the assessment of femoral version [59].

#### 2.2.2. Knee Joint Q-Angle Assessment

The Q-angle measurement is typically used to assess the degree of quadriceps muscle alignment to the underlying associated skeletal structures, in particular, the knee joint [60]. This measure has shown fair to moderate reliability (ICC 0.35–0.42) in children aged 9 to 16 years [61] and high reliability (ICC: 0.95) in young athletes [53]. The Q-angle was measured in an upright weight-bearing position with the knee fully extended using a standard goniometer with the reference arm pointed towards anterior superior iliac spine and the mobile arm on the tibial tuberosity to measure the angle between anterior superior iliac spine and tibial tuberosity [60].

#### 2.2.3. Tibial Torsion

Tibial torsion has been reported in children and adolescents [53] with moderate intrarater and interrater reliability (ICC: 0.86–0.87) and was measured in a supine position with knees kept neutral to avoid medial or lateral deviation. A gravity goniometer (Company ICB, Kirrawee, NSW, Australia, ICB gravity goniometer) which points to both the medial and lateral malleoli was used to measure the transmalleolar angle as previously described [62,63].

#### 2.2.4. Subtalar Joint Inversion and Eversion Range of Movement

The measurement of subtalar joint (STJ) inversion and eversion was performed by locating the subtalar joint neutral position with the participant in a prone, non-weight-bearing position. The goniometric measurement of ankle dorsiflexion showed good reliability in children and adults with orthopaedic conditions [64,65]. The leg-calcaneal angle was measured when the ankle was moved medially (heel maximally inverted) and laterally by aligning the goniometer axis with the malleoli, the mobile arm with the posterior midline of calcaneus, and the reference arm with the posterior midline of the lower leg [64].

#### 2.2.5. Foot Posture Index

The FPI (https://pmc.ncbi.nlm.nih.gov/articles/PMC2553778/ accessed on 20 February 2022) is an observational instrument designed to measure foot posture in a clinical setting in a weight-bearing position [66]. The FPI is reported to have good reliability in children [67,68,69] and has been validated in paediatric populations [70]. This clinical instrument consists of six items that refer to the forefoot and hindfoot positions of a child in an upright, relaxed stance, as well as the three dimensions of motion. The rearfoot was assessed by talar head palpation; observation of lateral malleolar curvature as well as the extent of inversion/eversion of the calcaneus. The forefoot assessment comprises measuring the talonavicular joint protrusion, medial longitudinal arch congruence a well as the abduction/adduction extent of the forefoot on the rearfoot [66]. The FPI score ranges from −12 (highly supinated) to +12 (highly pronated).

### 2.3. Statistical Analysis

Sample size calculation was performed using a Cohen’s d of 0.8, where a sample size of 26 participants per group provided 80% power to detect a statistically significant difference at the 5% significance level. Statistical analyses were performed using R version 4.3.2 (31 October 2023 ucrt) (R Foundation for Statistical Computing, Vienna, Austria). Demographic variables were summarised using median, range (min and max), and interquartile range (IQR, quartile 1 and quartile 3). Height, weight, BMI, sex, and age-adjusted percentiles were obtained from the Centre for Disease Control and Prevention (CDC), May 2000 data, using the R package cdcanthro Version, 0.1.0 [71]. Differences between children with and without GJH were assessed with Wilcoxon rank sum test for continuous variables and chi-square test for categorical data.

Differences in lower limb biomechanical and anthropometric metrics were compared between children with and without GJH in the whole sample and in 4 subsets: males, females, 11 years old or under and 12 years old or over. Median, range (min and max), and IQR were used to summarise these metrics. The association between GJH and non-GJH with lower limb biomechanical and anthropometric metrics was assessed with the Wilcoxon rank sum test. The median difference and 95% confidence interval (CI) for the difference were computed from the Hodges–Lehmann statistics [72]. If data subsets were skewed and contained small sample sizes non-parametric statistical tests were used with the median and interquartile range as robust descriptions of the data [72,73].

## 3. Results

### 3.1. Study Participant Characteristics

The anthropometric characteristics of all study participants are summarised in Table 3 and according to sex in Table 4. Overall, 25 participants were recruited in the hypermobility group and 27 in the control group. Data collection was completed over a 12-month period at a single time point for each participant. The median Beighton score for the hypermobile participants was 6 (range 5 to 9), and controls was 0 (range 0–1). There were no significant differences in age, sex, or height, weight and BMI percentiles of children with and without GJH.

### 3.2. Biomechanical Profiles

**Overall GJH vs. Control** (Table 5). All biomechanical outcome measures were significantly greater for participants with GJH (*p* < 0.001) with the exception of tibial torsion. For the GJH group, this metric showed significantly lower tibial torsion for right (*p* = 0.02) and non-significantly for left (*p* = 0.067) compared to the control group. The largest difference between children with and without GJH was related to the ankle ROM [right: median difference: 9 (95%CI: 7,12); left: median difference: 9 (95%CI: 6,12)], followed by the internal hip rotation [right and left: median difference: 7 (95%CI: 5,9)].

**Within sex comparisons** (Table 6). Compared to males, females with GJH showed higher differences in the medians for all biomechanical measures in comparison to non-GJH peers. In particular, tibial torsion, both left and right, showed a larger difference in medians in females than in males with GJH.

**Female GJH vs. control vs. male GJH vs. control** (Table 6). While there was no significant difference for tibial torsion in males, there was significantly greater internal tibial torsion in females with GJH compared to non-GJH peers [right: median difference: −4 (95% CI:−7,−2), *p* = 0.002, left: median difference: −5 (95%CI: −7,−1), *p* = 0.010]. In addition, changes in left internal hip rotation measure in males with GJH were not statistically significant compared to non-GJH peers [median difference:6 (95% CI: 0,11), *p* = 0.062].

**Age comparisons** (Table 7). In comparing the biomechanical characteristics between children with and without GJH aged 11 years or younger and 12 years or older, all measures except for tibial torsion were statistically significantly higher for children with GJH in both age groups. The difference in the medians for all biomechanical measures, except FPI scores, was comparable between the two age groups. In participants with GJH aged 12 years and over FPI median difference for the right and left foot were 7 (95% CI: 4,9) and 8 (95% CI: 4,10) while for 11 years or younger group median difference were 5 (95% CI: 3,6) and 4 (95%CI: 3,6), respectively.

## 4. Discussion

The current study was designed to profile the differences in the lower limb biomechanical parameters between children with and without GJH in order to inform clinical practice and facilitate the development of targeted therapy recommendations for this cohort. Our research outcomes validate our hypothesis, revealing a clear disparity in biomechanical observations between children exhibiting hypermobility and those without this condition. Overall, all biomechanical measures (except tibial torsion) were significantly higher for children with GJH compared to non-GJH peers. In addition, only females with GJH showed significantly greater internal tibial torsion compared to non-GJH females.

### 4.1. Overview of Biomechanical Profiles

In line with previous studies, no clear differences in anthropometric profiles were observed between children with GJH and their non-GJH peers [24,25,74]. However, there were major differences in the majority of biomechanical outcome measures for the lower limb between these two groups, including internal hip rotation, Q angle, ankle ROM, and FPI scores. Except for internal tibial torsion, all other measures were significantly higher in children with GJH, especially among females, compared to children without GJH. This study is the first to examine a wide range of lower limb biomechanical features, in particular Q-angle and FPI measurements, in children and adolescents with GJH. Therefore, these findings present early data to contribute towards establishing reference values for future investigations of this cohort.

### 4.2. Comparison with Previous Studies

#### 4.2.1. Significance of Internal Hip Rotation in Children with GJH

Children and adolescents with hypermobility demonstrated greater internal hip rotation compared to non-GJH children, similar to findings from previous studies in children [75] and adults [76]. In a prospective study, young children with GJH aged 7 to 13 years were shown to have significantly higher scores for internal hip rotation compared to non-hypermobile peers [75]. However, in a study evaluating GJH association with ROM in young female dancers and non-dancers aged 8 to 16 years, no significant differences in internal hip rotation measured according to Magee technique [77] were found between girls with and without GJH [29]. This is in contrast to our study, where females with GJH of all ages (5- 18 years) showed significantly higher values for internal hip rotation. One possible explanation might be due to methodological differences in the measurement of reference positions. We used the validated Craig’s Test to measure internal hip rotation with the most lateral aspect of the greater trochanter as the reference landmark [57], whereas in the dancers’ study, the axis of the goniometer was placed at the mid-patella area [29]. Another reason could be the use of different Beighton score cut-offs, we used ≥6/9 for prepubertal children and ≥5/9 for pubertal, whereas the dancers’ study used ≥6/9 across all ages of 8–16 years [29]. Although a recent meta-analysis recommended a Beighton score of ≥6/9 for children up to 18 years of age, due to limited available data and inconsistent reporting among included studies, pubertal status was not evaluated [78]. Thus, future studies should investigate the impact of pubertal status on Beighton score when comparing the biomechanical characteristics between children with and without GJH. While the clinical significance of the change in hip internal rotation in children with GJH may be unclear at the present time, nevertheless, our results are important for understanding anatomical differences in this cohort compared to healthy children.

#### 4.2.2. Q-Angle Values and Their Implications in Children with GJH

In our study, the Q-angle values were found to be 1.5–1.7 times greater in children and adolescents with hypermobility than in non-hypermobile controls. Similarly, in other studies, in college students [60,79] and young athletes [80,81] with GJH, higher values of Q-angle were significantly associated with hypermobility. In a cross-sectional study of healthy male college students (20–28 years), higher Q-angle measurements were consistently associated with increasing Beighton score values [60]. Likewise, in a more recent study of young students (18–30 years), in the relaxed standing position, the Q-angle on the non-dominant side was found to be significantly higher in the GJH group compared to the non-GJH group [79]. Significant correlation between higher Beighton score and Q-angle in competitive athletes (mean age: 21 years) has also been reported [80]. In addition, the Q-angle values were found to be greater in female athletes (mean age: 23 years) with higher Beighton scores [81].

Collectively, these studies support our findings that children and adolescents with GJH are at increased risk of injuries [11,36,37,38,39], Q-angle may have prognostic value for possible knee injury and should be considered as an integral part of clinical assessment for this cohort. In one previous study, in contrast to the findings of this study, no significant differences were reported in the Q-angle of young children (7–13 years) with and without hypermobility [75]. The key difference was that in our study, children with hypermobility showed excessive pronation, while in the previous study, the foot posture angular changes were within the normal range for the children with GJH [75]. This is an important consideration since an association between Q-angle and foot posture has been proposed, where abnormal foot posture can potentially impact the magnitude of Q-angle [82,83,84,85]. However, there is currently limited evidence, and more research is needed to clarify the role of foot posture and variations in Q-angle magnitude [86]. Another possible reason for the difference in Q-angle findings might be due to the use of different Beighton score cut-offs. In the previous study, children with GJH were identified with a cut-off score of ≥5/9 for females and ≥4/9 scores for males [87], whereas we used the higher threshold of ≥6/9 for prepubertal children and ≥5/9 for pubertal. A recent meta-analysis of worldwide prevalence data concluded that a Beighton score of ≥6/9 for identifying GJH in children should be used as a bare minimum and that a Beighton score of ≥7/9 should be considered for females [78].

Also, differences in the methodology of Q-angle measurement may account for discrepancies with our study, as individuals with hypermobility are particularly sensitive to the temporary correction of body posture [88]. In response to balance perturbations, adults with GJH (mean age: 28 years, Beighton score: ≥4/9) have been found to have higher cumulative joint angle values than adults with normal flexibility, indicating increased instability [88]. Nevertheless, further studies of biomechanical assessments in hypermobile children diagnosed with the current recommended Beighton score classification are warranted.

#### 4.2.3. Role of Ankle ROM in the Assessment of Children with GJH

Consistent with our findings, in a study comparing young dancers to non-dancers with and without GJH (8–16 years), the ankle ROM (inversion and eversion) was greater in children with GJH than in non-hypermobile matched controls [89]. While measuring ankle ROM in individuals with hypermobility has been shown to be useful for quantitative characterisation of foot kinematics during gait [23] and biomechanical gait analysis in children with hypermobility [24] to identify and develop rehabilitative strategies, the importance of ankle ROM measurement as part of clinical assessment in children with GJH remains to be further explored.

#### 4.2.4. Correlation Between FPI Scores and Hypermobility

We found a significantly higher FPI indicating excessive pronation in children with hypermobility compared to non-hypermobile peers. In line with the current findings, previous studies have shown a correlation between excessive pronation and hypermobility [28,47,48,90,91]. In a convenience sample of healthy asymptomatic children, greater FPI scores were associated with higher Beighton scores [28]. Since forces on foot alignment can greatly influence lower limb alignment, as demonstrated in induced hyperpronation in healthy adults during standing, which resulted in immediate internal hip rotation [31], foot posture assessment, together with internal hip rotation and Q-angle measures, should be considered as part of biomechanical measures in children with hypermobility.

#### 4.2.5. Tibial Torsion in Hypermobile Children

Overall, compared to the controls, the tibial torsion was not significantly different in hypermobile children. However, the sex analyses showed that this metric was significantly different for females but not males. This is in contrast to recent studies reporting no significant association for higher Beighton score and tibial torsion in GJH compared to non-GJH paediatric population [75,92]. However, these studies only reported tibial torsion in younger children aged 5–13 years old and used the Thigh-Foot Angle (TFA) test for measurement, whereas we measured the transmalleolar axis (TMA), which is largely used to measure tibial torsion. While TFA measures the angle between the longitudinal thigh-foot axis, the TMA measures the angle between the longitudinal thigh axis to the most prominent points on the medial–lateral malleolus axis [63]. For measurement of tibial torsion, TMA has been found to have greater validity and reliability compared to TFA and CT imaging [93]. To explore the variability of tibial torsion, our findings need to be confirmed with further controlled trials of sufficient sample size of both sexes and different age groups, comparing different test assessment tools. Collectively, these potential variations in biomechanical features of children and adolescents with GJH may result in unfavourable mechanical forces that can increase joint stress and lead to a higher risk of musculoskeletal injuries.

### 4.3. Clinical Implications and Potential Impact of Biomechanical Variations in Children with GJH

From a clinical perspective, given that GJH in children is associated with an increased risk of musculoskeletal injuries such as joint sprains, dislocations, and soft tissue injuries, particularly in weight-bearing joints such as the knees and ankles [11,19,36,94], the characterisation of biomechanical factors in children with GJH may be relevant for early identification of at risk children to prevent or reduce such musculoskeletal injuries through developing early preventative training programmes and tailored physical activity therapies.

This increased risk of injuries in children with GJH is likely due to the decreased joint stability and altered biomechanics often associated with joint hypermobility, making these children more susceptible to injury during physical activities. In addition, there is growing evidence that many hypermobile individuals exhibit a higher FPI score, which indicates a tendency toward pronation or flat feet [47,48,90,91]. A higher FPI score is common among hypermobile patients and can contribute to pain and instability in the feet and lower limbs [95]. This alignment issue in the feet further exacerbates the biomechanical challenges faced by those with hypermobility, affecting gait and posture and leading to discomfort in daily activities. Furthermore, the lower limbs-particularly the knees, ankles, and feet-are the most commonly reported areas of pain and dysfunction among hypermobile patients [8]. This is consistent with clinical observations that children with hypermobility frequently experience symptoms like pain, fatigue, and instability in these regions [7,8,10].

Altered joint moments have been reported in children with hypermobility who often present with increased joint ROM, leading to poor dynamic balance [49,50]. Foot orthoses have been shown to positively impact gait patterns and improve balance, pain, and function in children with hypermobility [49,51,52]. Clinicians should recognise the impact of these anatomical variations and screen for a range of lower extremity biomechanical parameters to identify children with GJH at risk of injuries and implement appropriate physical activity approaches.

### 4.4. Limitations

The findings of this study should be interpreted with due consideration. Firstly, as this was a cross-sectional study and no joint kinetics, kinematics, or gait analysis was conducted, no assumptions should be made about cause–effect relationships. Since few studies have investigated the correlation between kinematic and temporal-spatial gait with internal hip rotation in children with hypermobility [96,97,98], this relationship remains to be further explored in larger controlled trials. While this is an exploratory study to examine multiple lower limb biomechanical characteristics between children with GJH and non-GJH, it is limited to a relatively small sample size (n = 52), and, therefore, these values may not be adequately representative of the population at large, and caution should be exercised to consider these values as normative data for children with hypermobility. Secondly, sex distribution was unbalanced as 72% of the GJH children were girls in this study, which reflects the higher frequency of GJH among females [99,100]. A recent systematic review in children demonstrated a statistically significantly higher prevalence of GJH (Beighton score ≥ 6/9) in females compared to males with a mean difference of 7.3% [78]. This sex disparity was reflected in our recruitment process, where we observed a higher proportion of female participants. While this imbalance was not unexpected, it may have influenced the outcomes of our study. Future research could benefit from efforts to recruit a more balanced male-to-female ratio or from exploring sex-specific factors that may contribute to differences in hypermobility. In addition, generalisability outside this population is limited since children with symptomatic hypermobility were excluded, as pain may have impacted the results. Furthermore, we could not evaluate the physical activity level, history of injuries, and functional problems of the included participants. There was also a lack of studies on the biomechanical assessment of children with GJH, with no study comparing variations in FPI together with Q-angle in adolescents. Due to paucity of data in children, we drew support for our findings from studies in young adults with GJH (≥18 years) for Q-angle measures, which have been shown to stabilise in young football players aged 21 years and over [101].

It is also important to acknowledge the measurement error and clinical significance of our data when interpreting the findings of this study. Some of the measurement errors for the biomechanical outcomes were large, which might indicate increased variability, reduced reliability of data, and undermine statistical power to draw definitive conclusions. To reduce random error and minimise its impact on the final data, the number of measurements was repeated three times and the average calculated. Furthermore, in order to reduce variability, one investigator completed all measurements and followed standardised protocols. Considering the current advances in digital technologies for biomechanical assessments, such as smartphone clinometer applications validated for measurements of hip rotation [102] and digital goniometer to assess joint ROM in the lower limb [103], such technologies can be used to reduce the level of error in measurements and improve the accuracy of data. While median values are significantly higher for most of the biomechanical measures in children with GJH compared to non-GJH peers, some of these differences between significant and non-significant were as small as 4 degrees, and it is unclear whether this translates to clinical relevance. Therefore, whether this “significance” is clinically meaningful needs to be interpreted carefully, and the clinical relevance of our findings confirmed in future longitudinal studies.

The broad age range of participants (5–18 years) also presents an important limitation, as developmental differences can affect biomechanical outcomes due to variations in growth and skeletal development. To mitigate this, we matched participants by age and sex within a one-year range, ensuring comparable biomechanical comparisons.

The complex inter-relationship between the high FPI score, increased Q-angle, and internal hip rotation also warrants further consideration. Specifically, an increased Q-angle can contribute to greater internal hip rotation, and conversely, internal hip rotation can influence the alignment of the knee and lower leg, potentially leading to altered foot mechanics, as captured by the FPI. Moreover, the link between increased internal hip rotation and external tibial torsion is another area that could benefit from deeper exploration. These interconnected biomechanical factors can compound the overall misalignment, contributing to compensatory movements that may exacerbate symptoms in hypermobile individuals. By investigating these relationships more thoroughly, we can gain a better understanding of how each factor contributes to the overall biomechanical profile and symptoms in this population. Further longitudinal prospective studies in larger samples of children and adolescents exploring these complex interactions and evaluating a range of common biomechanical measures are warranted. In addition, controlled studies on joint kinematics are needed to support the causality between these variations in joint alignment and hypermobility.

## 5. Conclusions

This research represents the first age and sex-matched case–control study to identify biomechanical differences in children and adolescents with generalised joint hypermobility (GJH) for lower extremity joints, specifically Foot Posture Index (FPI) and tibial torsion. We proposed that children with hypermobility exhibit a distinct biomechanical profile compared to their peers without hypermobility. The current study confirmed this hypothesis. The main difference between children with and without GJH was in ankle ROM and internal hip rotation. Females with GJH showed greater median differences in biomechanical measures than males. While females with GJH had significantly greater internal tibial torsions compared to non-GJH peers, males showed no significant difference. In both age groups (≤11 and ≥12 years), children with GJH had significantly higher measures except tibial torsion, with similar median differences across age groups for all measures except FPI scores. The findings further indicated that the Q-angle and FPI measures were greater in adolescents with GJH than in their healthy peers, possibly suggesting a higher susceptibility to injuries among this group of children. While findings highlight that the majority of lower limb biomechanical measurements were significantly altered in GJH participants compared to their non-hypermobile peers, the clinical relevance of these values needs further in-depth exploration in prospective analyses. Nonetheless, considering the range of symptoms, and particularly the lower limb problems experienced by children affected with hypermobility, this study highlights the importance of assessment of lower limb hypermobility using a range of biomechanical measures together to overcome the limitation of the Beighton score and therefore enhance clinical practice outcomes for this cohort. Based on these preliminary findings, further controlled studies with larger samples are warranted to examine the potential impact of these biomechanical differences in response to exercise recommendations.

## Figures and Tables

**Table 1:** Eligibility criteria.

Inclusion Criteria	Exclusion Criteria
Aged between 5 and 18 years Adequate vision, hearing, and cognitive ability to understand instructions Speak English to consent to biomechanical assessment Beighton score for children diagnosed with hypermobility: *≥5 pubertal* *≥6 pre-pubertal*	>18 years old Lower limb pain, swelling or acute injuries Inability to walk unassisted Unable to consent or understand instructions Presence of concomitant musculoskeletal disease, central or peripheral nerve disease

**Table 2:** Summary of biomechanical assessments using goniometer for lower extremities.

Assessments	Participant Position	Goniometer		
		Reference Arm	Mobile Arm	Axis
**Internal hip Rotation** **(Craig’s Test)**	Prone	Perpendicular to floor (representing femoral neck axis)	Parallel to tibia (representing line between femoral condyles)	Most lateral aspect of greater trochanter
**Knee joint assessment** **(Q angle)**	Upright	Pointed to ASIS	on tibial tuberosity	Centre of patella
**Tibial torsion** **(transmalleolar angle)**	Supine	Medial and lateral malleoli (transmalleolar axis)	longitudinal axis of the thigh (transcondylar axis)	transmalleolar axis
**Subtalar Joint Inversion and Eversion**	Prone	Posterior midline of lower leg	Posterior midline of calcaneus	Posterior aspect of subtalar joint midway between malleoli

**Abbreviations:** ASIS: anterior superior iliac spine; Q angle: Quadriceps angle.

**Table 3:** Demographic summary of the total sample by generalised joint hypermobility (GJH).

Characteristic	GJH N = 25 ^1^	Non-GJH N = 27 ^1^	*p*-Value ^2^	Overall N = 52 ^1^
**Age**			0.580	
**Median (IQR)**	11.00 (9.00, 12.00)	11.00 (9.00, 14.00)		11.00 (9.00, 13.00)
**Range**	5.00, 18.00	6.00, 18.00		5.00, 18.00
**Sex**			0.677	
**Female**	18 (72%)	18 (67%)		36 (69%)
**Male**	7 (28%)	9 (33%)		16 (31%)
**Beighton score**			<0.001	
**Median (IQR)**	6.00 (6.00, 8.00)	0.00 (0.00, 0.00)		1.00 (0.00, 6.00)
**Range**	5.00, 9.00	0.00, 1.00		0.00, 9.00
**Height-sex age percentile**			0.088	
**Median (IQR)**	69 (32, 85)	46 (34, 50)		47 (33, 69)
**Range**	0, 95	9, 100		0, 100
**Weight-sex age percentile**			0.048	
**Median (IQR)**	68 (38, 85)	47 (41, 50)		49 (41, 70)
**Range**	0, 98	33, 92		0, 98
**BMI-sex age percentile**			0.139	
**Median (IQR)**	65 (32, 87)	52 (47, 58)		54 (47, 73)
**Range**	0, 100	34, 80		0, 100

^1^ n (%). ^2^ Wilcoxon rank sum test; Pearson’s Chi-squared test; Wilcoxon rank sum exact test.

**Table 4:** Demographics summary of the sample by generalised joint hypermobility (GJH) and sex.

Characteristic	Hyper N = 7	Non-Hyper N = 9	*p*-Value ^1^	Overall N = 16	Hyper N = 18	Non-Hyper N = 18	*p*-Value ^1^	Overall N = 36
*Males*					***Females***			
**Age**			0.589				0.408	
**Median (IQR)**	12.00 (10.50, 12.00)	10.00 (9.00, 12.00)		11.00 (9.00, 12.00)	10.00 (9.00, 13.00)	12.50 (9.25, 14.00)		11.00 (9.00, 14.00)
**Range**	7.00, 12.00	7.00, 16.00		7.00, 16.00	5.00, 18.00	6.00, 18.00		5.00, 18.00
**Height-sex age percentile**			0.916				0.035	
**Median (IQR)**	75 (15, 91)	47 (44, 50)		47 (41, 79)	65 (37, 77)	44 (31, 50)		48 (33, 63)
**Range**	0, 95	9, 100		0, 100	6, 91	14, 57		6, 91
**Weight-sex age percentile**			0.351				0.060	
**Median (IQR)**	86 (46, 92)	48 (47, 61)		56 (45, 87)	61 (39, 81)	45 (41, 49)		48 (41, 66)
**Range**	0, 96	33, 92		0, 96	0, 98	37, 66		0, 98
**BMI-sex age percentile**			0.681				0.104	
**Median (IQR)**	86 (33, 88)	54 (53, 61)		55 (44, 81)	65 (36, 83)	49 (47, 57)		51 (47, 67)
**Range**	6, 100	36, 80		6, 100	0, 96	34, 74		0, 96

^1^ Wilcoxon rank sum test.

**Table 5:** Associations of characteristics with generalised joint hypermobility (GJH) ^†^.

Characteristic	Overall N = 52	GJH N = 25	Non-GJH N = 27	Median Difference (95%CI)	*p*-Value ^1^
***Craig’s test (internal rotation) RIGHT***					<0.001
**Median (IQR)**	23.5 (20.0, 26.0)	26.0 (25.0, 30.0)	20.0 (17.5, 23.0)	7 (5, 9)	
**Range**	12.0, 35.0	18.0, 35.0	12.0, 26.0		
***Craig’s test (internal rotation) LEFT***					<0.001
**Median (IQR)**	23.0 (20.0, 27.0)	27.0 (25.0, 31.0)	20.0 (18.5, 22.5)	7 (5, 9)	
**Range**	12.0, 34.0	17.0, 34.0	12.0, 26.0		
***Knee Position Extension Q angle RIGHT***					<0.001
**Median (IQR)**	9.50 (7.00, 12.00)	12.00 (11.00, 14.00)	8.00 (6.00, 8.00)	4 (3, 6)	
**Range**	4.00, 15.00	7.00, 15.00	4.00, 12.00		
***Knee Position Extension Q angle LEFT***					<0.001
**Median (IQR)**	9.50 (7.00, 12.00)	12.00 (10.00, 14.00)	7.00 (6.00, 8.00)	4 (3, 6)	
**Range**	4.00, 17.00	7.00, 17.00	4.00, 12.00		
***Tibial torsion (Transmalleolar angle) RIGHT***					0.020
**Median (IQR)**	14.0 (12.0, 17.0)	12.0 (8.0, 15.0)	15.0 (13.0, 17.0)	−3 (−5, −1)	
**Range**	4.0, 20.0	4.0, 20.0	12.0, 18.0		
***Tibial torsion (Transmalleolar angle) LEFT***					0.067
**Median (IQR)**	15.0 (12.0, 16.3)	14.0 (8.0, 16.0)	15.0 (14.0, 17.0)	−3 (−6, 0)	
**Range**	0.0, 21.0	0.0, 21.0	12.0, 20.0		
***Subtalar Joint ROM total RIGHT***					<0.001
**Median (IQR)**	33.0 (30.0, 40.3)	41.0 (36.0, 43.0)	30.0 (29.0, 32.0)	9 (7, 12)	
**Range**	28.0, 48.0	29.0, 48.0	28.0, 34.0		
***Subtalar Joint ROM total LEFT***					<0.001
**Median (IQR)**	33.0 (31.0, 39.3)	40.0 (35.0, 43.0)	31.0 (29.5, 32.0)	9 (6, 12)	
**Range**	27.0, 47.0	27.0, 47.0	28.0, 34.0		
***Subtalar Joint ROM inversion RIGHT***					<0.001
**Median (IQR)**	23.1 (21.0, 28.2)	28.7 (25.2, 30.1)	21.0 (20.3, 22.4)	6.3 (4.9, 8.4)	
**Range**	19.6, 33.6	20.3, 33.6	19.6, 23.8		
***Subtalar Joint ROM eversion RIGHT***					<0.001
**Median (IQR)**	9.90 (9.00, 12.08)	12.30 (10.80, 12.90)	9.00 (8.70, 9.60)	2.7 (2.1, 3.6)	
**Range**	8.40, 14.40	8.70, 14.40	8.40, 10.20		
***Subtalar Joint ROM inversion LEFT***					<0.001
**Median (IQR)**	23.1 (21.7, 27.5)	28.0 (24.5, 30.1)	21.7 (20.7, 22.4)	6.3 (4.2, 8.4)	
**Range**	18.9, 32.9	18.9, 32.9	19.6, 23.8		
***Subtalar Joint ROM eversion LEFT***					<0.001
**Median (IQR)**	9.90 (9.30, 11.78)	12.00 (10.50, 12.90)	9.30 (8.85, 9.60)	2.7 (1.8, 3.6)	
**Range**	8.10, 14.10	8.10, 14.10	8.40, 10.20		
***Foot Posture Index (FPI) RIGHT***					<0.001
**Median (IQR)**	6.5 (4.0, 9.0)	9.0 (8.0, 10.0)	4.0 (0.0, 5.0)	5 (4, 7)	
**Range**	−2.0, 12.0	0.0, 12.0	−2.0, 7.0		
***Foot Posture Index (FPI) LEFT***					<0.001
**Median (IQR)**	6.0 (4.0, 9.0)	9.0 (8.0, 10.0)	4.0 (0.0, 5.0)	5 (4, 7)	
**Range**	−2.0, 12.0	0.0, 12.0	−2.0, 7.0		

^1^ Wilcoxon rank sum test. ^†^ All values are in degrees except for FPI.

**Table 6:** Associations of characteristics with generalised joint hypermobility (GJH) according to sex ^†^.

Sex	Males					Females				
Characteristic	Overall N = 16	GJH N = 7	Non-GJH N = 9	Median Difference (95%CI)	*p*-Value ^1^	Overall N = 36	GJH N = 18	Non-GJH N = 18	Median Difference (95%CI)	*p*-Value ^1^
**Craig’s test (internal rotation) RIGHT**					0.040					<0.001
**Median (IQR)**	21.5 (20.0, 25.0)	25.0 (22.5, 30.0)	20.0 (20.0, 23.0)	5 (0, 10)		24.5 (19.5, 26.3)	26.5 (25.0, 29.8)	19.0 (17.3, 23.8)	7 (4, 10)	
**Range**	15.0, 32.0	18.0, 32.0	15.0, 23.0			12.0, 35.0	20.0, 35.0	12.0, 26.0		
**Craig’s test (internal rotation) LEFT**					0.062					<0.001
**Median (IQR)**	21.5 (20.0, 25.3)	26.0 (22.5, 30.5)	20.0 (20.0, 22.0)	6 (0, 11)		24.0 (20.0, 27.0)	27.0 (26.0, 30.8)	20.0 (18.0, 22.8)	7 (4, 10)	
**Range**	15.0, 34.0	17.0, 34.0	15.0, 23.0			12.0, 33.0	20.0, 33.0	12.0, 26.0		
**Knee Position Extension Q angle RIGHT**					0.006					<0.001
**Median (IQR)**	8.00 (7.00, 11.25)	12.00 (10.00,13.50)	7.00 (7.00, 8.00)	5 (2, 7)		10.00 (8.00, 12.00)	11.50 (11.00, 13.75)	8.00 (6.00, 9.75)	4 (3, 6)	
**Range**	4.00, 15.00	7.00, 15.00	4.00, 8.00			4.00, 15.00	8.00, 15.00	4.00, 12.00		
**Knee Position Extension Q angle LEFT**					0.012					<0.001
**Median (IQR)**	8.00 (7.00, 10.25)	11.00 (9.00, 13.50)	8.00 (6.00, 8.00)	5 (1, 7)		10.00 (7.00, 12.00)	12.00 (10.00, 14.00)	7.00 (6.00, 9.50)	4 (3, 6)	
**Range**	4.00, 15.00	7.00, 15.00	4.00, 8.00			4.00, 17.00	8.00, 17.00	4.00, 12.00		
**Tibial torsion (Transmalleolar angle) RIGHT**					0.423					0.002
**Median (IQR)**	16.0 (13.8, 18.0)	17.0 (14.5, 19.0)	16.0 (13.0, 16.0)	2 (−3, 5)		14.0 (10.0, 16.0)	10.0 (8.0, 14.0)	14.5 (13.3, 17.0)	−4 (−7, −2)	
**Range**	4.0, 20.0	4.0, 20.0	12.0, 18.0			6.0, 20.0	6.0, 20.0	12.0, 18.0		
**Tibial torsion (Transmalleolar angle) LEFT**					0.393					0.010
**Median (IQR)**	15.0 (14.0, 18.3)	15.0 (15.0, 19.5)	15.0 (14.0, 17.0)	1 (−3, 5)		14.0 (9.8, 16.0)	9.5 (8.0, 15.0)	15.0 (14.0, 16.8)	−5 (−7, −1)	
**Range**	0.0, 21.0	0.0, 21.0	13.0, 19.0			5.0, 20.0	5.0, 18.0	12.0, 20.0		
***Subtalar Joint* ROM total RIGHT**					0.022					<0.001
**Median (IQR)**	31.0 (29.0, 36.5)	38.0 (33.0, 38.5)	30.0 (28.0, 32.0)	7 (1, 10)		33.0 (30.8, 41.0)	41.0 (37.0, 44.8)	31.0 (29.0, 32.8)	11 (7, 13)	
**Range**	28.0, 41.0	29.0, 41.0	28.0, 33.0			28.0, 48.0	30.0, 48.0	28.0, 34.0		
***Subtalar Joint* ROM total LEFT**					0.009					<0.001
**Median (IQR)**	32.0 (30.8, 36.3)	37.0 (34.0, 38.5)	31.0 (29.0, 32.0)	6 (2, 9)		33.5 (31.0, 42.3)	42.5 (35.8, 44.5)	31.0 (30.0, 31.8)	11 (7, 13)	
**Range**	28.0, 40.0	31.0, 40.0	28.0, 34.0			27.0, 47.0	27.0, 47.0	28.0, 34.0		
***Subtalar Joint* inversion RIGHT**					0.022					<0.001
**Median (IQR)**	21.70 (20.30, 25.55)	26.60 (23.10, 26.95)	21.00 (19.60, 22.40)	4.9 (0.7, 7)		23.1 (21.5, 28.7)	28.7 (25.9, 31.3)	21.7 (20.3, 22.9)	7.7 (4.9, 9.1)	
**Range**	19.60, 28.70	20.30, 28.70	19.60, 23.10			19.6, 33.6	21.0, 33.6	19.6, 23.8		
***Subtalar Joint* ROM eversion RIGHT**					0.022					<0.001
**Median (IQR)**	9.30 (8.70, 10.95)	11.40 (9.90, 11.55)	9.00 (8.40, 9.60)	2.1 (0.3, 3)		9.90 (9.23, 12.30)	12.30 (11.10, 13.43)	9.30 (8.70, 9.83)	3.3 (2.1, 3.9)	
**Range**	8.40, 12.30	8.70, 12.30	8.40, 9.90			8.40, 14.40	9.00, 14.40	8.40, 10.20		
***Subtalar Joint* ROM inversion LEFT**				<0.001	0.009					<0.001
**Median (IQR)**	22.40 (21.53, 25.38)	25.90 (23.80, 26.95)	21.70 (20.30, 22.40)	4.2 (1.4, 6.3)		23.5 (21.7, 29.6)	29.8 (25.0, 31.2)	21.7 (21.0, 22.2)	7.7 (4.9, 9.1)	
**Range**	19.60, 28.00	21.70, 28.00	19.60, 23.80			18.9, 32.9	18.9, 32.9	19.6, 23.8		
***Subtalar Joint* ROM eversion LEFT**					0.009					<0.001
**Median (IQR)**	9.60 (9.23, 10.88)	11.10 (10.20, 11.55)	9.30 (8.70, 9.60)	1.8 (0.6, 2.7)		10.05 (9.30, 12.68)	12.75 (10.73, 13.35)	9.30 (9.00, 9.53)	3.3 (2.1, 3.9)	
**Range**	8.40, 12.00	9.30, 12.00	8.40, 10.20			8.10, 14.10	8.10, 14.10	8.40, 10.20		
**Foot Posture Index (FPI) RIGHT**					<0.001					<0.001
**Median (IQR)**	6.5 (4.0, 9.0)	9.0 (8.5, 9.5)	4.0 (3.0, 5.0)	5 (3, 8)		6.5 (3.5, 9.3)	9.5 (7.3, 10.0)	4.0 (0.0, 5.0)	6 (4, 8)	
**Range**	0.0, 10.0	8.0, 10.0	0.0, 7.0			−2.0, 12.0	0.0, 12.0	−2.0, 7.0		
**Foot Posture Index (FPI) LEFT**					<0.001					<0.001
**Median (IQR)**	6.0 (4.0, 8.3)	9.0 (8.0, 10.0)	4.0 (3.0, 5.0)	5 (3, 8)		6.0 (3.5, 9.3)	9.5 (7.3, 10.0)	4.0 (0.0, 5.0)	6 (4, 8)	
**Range**	0.0, 10.0	8.0, 10.0	0.0, 7.0			−2.0, 12.0	0.0, 12.0	−2.0, 6.0		

^1^ Wilcoxon rank sum test. ^†^ All values are in degrees except for FPI.

**Table 7:** Associations of characteristics with generalised joint hypermobility (GJH) for age groups of ≤11 years and ≥12 years ^†^.

Age Group	11 Years and Under					12 Years and Over				
Characteristic	Overall N = 28	GJH N = 14	Non-GJH N = 14	Median Difference (95%CI)	p-Value ^1^	Overall N = 24	GJH N = 11	Non-GJH N = 13	Median Difference (95%CI)	p-Value ^1^
**Craigs test (internal rotation) RIGHT**					<0.001					0.001
Median (IQR)	25.0 (20.0, 28.0)	28.0 (25.0, 30.0)	20.0 (20.0, 23.0)	7 (4, 10)		21.0 (17.8, 25.0)	25.0 (22.5, 28.0)	18.0 (17.0, 22.0)	7 (3, 10)	
Range	17.0, 35.0	20.0, 35.0	17.0, 26.0			12.0, 32.0	18.0, 32.0	12.0, 24.0		
**Craigs test (internal rotation) LEFT**					<0.001					0.003
Median (IQR)	25.0 (20.0, 27.3)	27.5 (26.0, 31.0)	20.5 (20.0, 23.0)	7 (4, 10)		21.5 (18.8, 26.0)	26.0 (22.5, 29.0)	20.0 (16.0, 22.0)	7 (3, 11)	
Range	17.0, 33.0	20.0, 33.0	17.0, 26.0			12.0, 34.0	17.0, 34.0	12.0, 24.0		
**Knee Position Extension Q angle RIGHT**					<0.001					0.003
Median (IQR)	10.00 (8.00, 12.00)	12.00 (11.00, 13.75)	8.00 (7.00, 8.00)	4 (3, 6)		8.50 (6.75, 11.25)	11.00 (9.50, 13.50)	7.00 (5.00, 8.00)	4 (2, 6)	
Range	4.00, 15.00	9.00, 15.00	4.00, 10.00			4.00, 15.00	7.00, 15.00	4.00, 12.00		
**Knee Position Extension Q angle LEFT**					<0.001					0.003
Median (IQR)	10.00 (7.75, 12.00)	12.00 (10.25, 14.00)	7.50 (7.00, 8.00)	4 (3, 6)		8.00 (6.00, 12.00)	12.00 (9.00, 13.50)	6.00 (6.00, 8.00)	4 (2, 7)	
**Range**	4.00, 17.00	9.00, 17.00	4.00, 10.00			4.00, 15.00	7.00, 15.00	4.00, 12.00		
**Tibial torsion (Transmalleolar angle) RIGHT**					0.111					0.052
Median (IQR)	13.0 (10.0, 15.3)	10.0 (8.5, 15.0)	13.5 (13.0, 16.0)	−3 (−6, 1)		15.5 (13.8, 18.0)	14.0 (9.5, 16.5)	17.0 (15.0, 18.0)	−3 (−7, 0)	
Range	7.0, 20.0	7.0, 20.0	12.0, 17.0			4.0, 20.0	4.0, 20.0	13.0, 18.0		
**Tibial torsion (Transmalleolar angle) LEFT**					0.100					0.336
Median (IQR)	14.5 (11.3, 16.0)	10.5 (8.0, 15.0)	15.0 (14.0, 16.0)	−4 (−7, 0)		15.0 (13.0, 18.0)	15.0 (9.0, 17.0)	17.0 (13.0, 18.0)	−2 (−6, 1)	
Range	5.0, 21.0	5.0, 21.0	12.0, 18.0			0.0, 20.0	0.0, 19.0	12.0, 20.0		
***Subtalar Joint* ROM total RIGHT**					<0.001					0.001
Median (IQR)	33.5 (31.0, 41.0)	41.0 (36.5, 42.5)	31.0 (29.0, 32.0)	9 (6, 12)		32.5 (29.8, 38.3)	39.0 (34.5, 43.5)	30.0 (29.0, 32.0)	9 (4, 13)	
Range	28.0, 48.0	30.0, 48.0	28.0, 34.0			28.0, 46.0	29.0, 46.0	28.0, 33.0		
***Subtalar Joint* ROM total LEFT**					<0.001					0.002
Median (IQR)	33.5 (31.0, 40.3)	40.5 (35.8, 42.8)	31.0 (30.0, 32.5)	9 (5, 12)		32.0 (29.8, 37.3)	38.0 (35.5, 43.0)	31.0 (29.0, 32.0)	9 (4, 13)	
Range	28.0, 47.0	32.0, 47.0	28.0, 34.0			27.0, 46.0	27.0, 46.0	28.0, 34.0		
***Subtalar Joint* ROM inversion RIGHT**					<0.001					0.001
Median (IQR)	23.5 (21.7, 28.7)	28.7 (25.6, 29.8)	21.7 (20.3, 22.4)	6.3 (4.2, 8.4)		22.8 (20.8, 26.8)	27.3 (24.2, 30.5)	21.0 (20.3, 22.4)	6.3 (2.8, 9.1)	
Range	19.6, 33.6	21.0, 33.6	19.6, 23.8			19.6, 32.2	20.3, 32.2	19.6, 23.1		
***Subtalar Joint* ROM eversion RIGHT**					<0.001					0.001
Median (IQR)	10.05 (9.30, 12.30)	12.30 (10.95, 12.75)	9.30 (8.70, 9.60)	2.7 (1.8, 3.6)		9.75 (8.93, 11.48)	11.70 (10.35, 13.05)	9.00 (8.70, 9.60)	2.7 (1.2, 3.9)	
Range	8.40, 14.40	9.00, 14.40	8.40, 10.20			8.40, 13.80	8.70, 13.80	8.40, 9.90		
***Subtalar Joint* ROM inversion LEFT**					<0.001					0.002
Median (IQR)	23.5 (21.7, 28.2)	28.4 (25.0, 29.9)	21.7 (21.0, 22.8)	6.3 (3.5, 8.4)		22.4 (20.8, 26.1)	26.6 (24.9, 30.1)	21.7 (20.3, 22.4)	6.3 (2.8, 9.1)	
Range	19.6, 32.9	22.4, 32.9	19.6, 23.8			18.9, 32.2	18.9, 32.2	19.6, 23.8		
***Subtalar Joint* ROM eversion LEFT**					<0.001					0.002
Median (IQR)	10.05 (9.30, 12.08)	12.15 (10.73, 12.83)	9.30 (9.00, 9.75)	2.7 (1.5, 3.6)		9.60 (8.93, 11.18)	11.40 (10.65, 12.90)	9.30 (8.70, 9.60)	2.7 (1.2, 3.9)	
Range	8.40, 14.10	9.60, 14.10	8.40, 10.20			8.10, 13.80	8.10, 13.80	8.40, 10.20		
**Foot Posture Index (FPI) RIGHT**					<0.001					<0.001
Median (IQR)	7.00 (4.00, 9.00)	9.00 (7.25, 10.00)	4.00 (4.00, 5.75)	5 (3, 6)		5.0 (0.0, 9.0)	9.0 (8.0, 10.0)	2.0 (0.0, 5.0)	7 (4, 9)	
Range	0.00, 12.00	7.00, 12.00	0.00, 7.00			−2.0, 12.0	0.0, 12.0	−2.0, 5.0		
**Foot Posture Index (FPI) LEFT**					<0.001					<0.001
Median (IQR)	7.00 (4.00, 9.00)	9.00 (7.25, 10.00)	4.00 (4.00, 5.75)	4 (3, 6)		5.0 (0.0, 9.3)	10.0 (8.0, 10.0)	2.0 (0.0, 5.0)	8 (4, 10)	
Range	0.00, 12.00	7.00, 12.00	0.00, 7.00			−2.0, 12.0	0.0, 12.0	−2.0, 6.0		

^1^ Wilcoxon rank sum test. ^†^ All values are in degrees except for FPI.

## Data Availability

Additional data can be obtained from the corresponding author upon reasonable request.
